# Screening and Antifungal Activity of a *β*-Carboline Derivative against *Cryptococcus neoformans* and *C. gattii*

**DOI:** 10.1155/2019/7157845

**Published:** 2019-01-22

**Authors:** Kátia Santana Cruz, Emerson Silva Lima, Marcia de Jesus Amazonas da Silva, Erica Simplício de Souza, Andreia Montoia, Adrian Martin Pohlit, João Vicente Braga de Souza

**Affiliations:** ^1^Programa de Pós-Graduação da Rede de Biodiversidade e Biotecnologia da Amazônia Legal—Bionorte, Manaus, Amazonas, Brazil; ^2^Universidade Federal do Amazonas, Manaus, Amazonas, Brazil; ^3^Fundação de Medicina Tropical Heitor Vieira Dourado, Manaus, Amazonas, Brazil; ^4^Universidade Estadual do Amazonas, Manaus, Amazonas, Brazil; ^5^Instituto Nacional de Pesquisas da Amazônia, Manaus, Amazonas, Brazil

## Abstract

**Background:**

Cryptococcosis is a fungal disease of bad prognosis due to its pathogenicity and the toxicity of the drugs used for its treatment. The aim of this study was to investigate the medicinal potential of carbazole and *β*-carboline alkaloids and derivatives against *Cryptococcus neoformans* and *C. gattii*.

**Methods:**

MICs were established in accordance with the recommendations of the Clinical and Laboratory Standards Institute for alkaloids and derivatives against *C. neoformans* and *C. gattii* genotypes VNI and VGI, respectively. A single active compound was further evaluated against *C*. *neoformans* genotypes VNII, VNIII, and VNIV, *C*. *gattii* genotypes VGI, VGIII, and VGIV, *Candida albicans* ATCC 36232, for cytotoxicity against the MRC-5 lineage of human fibroblasts and for effects on fungal cells (cell wall, ergosterol, and leakage of nucleic acids).

**Results:**

Screening of 11 compounds revealed 8-nitroharmane as a significant inhibitor (MIC 40 *μ*g/mL) of several *C. neoformans* and *C. gattii* genotypes. It was not toxic to fibroblasts (IC_50_ > 50 *µ*g/mL) nor did it alter fungal cell walls or the concentration of ergosterol in *C. albicans* or *C. neoformans*. It increased leakage of substances that absorb at 260 nm.

**Conclusions:**

The synthetic *β*-carboline 8-nitroharmane significantly inhibits pathogenic *Cryptococcus* species and is interesting as a lead compound towards new therapy for *Cryptococcus* infections.

## 1. Introduction

Cryptococcosis, a fungal disease caused by *Cryptococcus neoformans* and *C. gattii*, occurs predominantly in immunocompromised individuals. *Cryptococcus* infections occur through inhalation of blastospores and basidiospores that establish a pulmonary infection. These infections can also disseminate to the meninges and brain, causing meningitis or meningoencephalitis [1]. The global incidence and impact of cryptococcosis (cryptococcal disease) is estimated to be 624,700 deaths annually, with a broad confidence interval of 125,000 to 1,124,900 [2].

For cryptococcosis treatment, the World Health Organization (WHO) recommends a two-week course of amphotericin B and flucytosine (5-FC) as the initial intensive induction phase and subsequently followed by a step-down to fluconazole for the consolidation and monitoring phases of treatment for cryptococcal meningitis [3]. The arsenal of treatment options available presently for management is limited, with no new class of antifungal agent exhibiting cryptococcal activity licensed in almost 30 years [4]. Treatment failure and drug toxicity are frequently observed, and the search for potential drugs for therapy is necessary [5].

Previous work has shown that harman alkaloids (Table 1) and related *β*-carbolines (*β*Cs) exhibit important antifungal properties. Harmol is fungicidal to *Botrytis cinerea* and fungistatic to *Penicillium digitatum* affecting conidia membrane permeabilization in both species [6]. Harmane selectively inhibited *Cercospora arachidicola* while its synthetic derivative 8-nitroharmane strongly inhibited 13 out of 14 fungi species tested [7]. A variety of synthetic *β*C analogs have recently been patented as fungicides for the treatment of plant diseases [8]. Of medicinal relevance, harmane [9] and harmine [10] are reported to be potent *Candida albicans* inhibitors and harmine in binary combinations with other *β*Cs strongly inhibits *Aspergillus niger*. Harmaline significantly inhibits *Candida rugosa* lipase *in vitro* as a competitive inhibitor according to *in silico* (docking) studies [11]. A recent patent describes the enhancement by harmine hydrochloride of the action of fluconazole against drug-resistant *C*. *albicans* in compositions that claim to reverse resistance to fluconazole [12]. These and other examples attest to the potential of harman *β*Cs of natural or synthetic origin as antifungal agents for medicinal and other applications.

To our knowledge, harman alkaloids or their derivatives have not been previously assayed against *Cryptococcus* spp. The aim of this study was to evaluate the antifungal activity of carbazoles and *β*Cs against the clinically relevant species *Cryptococcus neoformans* and *C. gattii*.

## 2. Materials and Methods

### 2.1. Microorganisms and Reference Strain

Eight lyophilized standard culture strains of *C. neoformans* (WM148/08; WM626/08; WM628/08; WM629/08) and *C. gattii* (WM179/08; WM178/08; WM179/08; WM779/08) were kindly provided by the Oswaldo Cruz Foundation (Fiocruz) in Rio de Janeiro, Brazil. *Candida albicans* strain ATCC 36232 from the culture collection at the National Institute for Amazonian Research (INPA) in Manaus, Amazonas state, Brazil, was used as reference.

The strains were reactivated in Sabouraud agar dextrose (SAD). An inoculum was removed from the SAD culture and suspended in 5.0 mL of sterile 0.085% saline water and vortexed for 15 s. The cell density was adjusted to 0.5 on the McFarland scale (comparison to reference).

### 2.2. Substances

Eleven commercial and synthetic carbazole and *β*-carboline derivatives (Table 1) were screened in this work. Prior to testing, substances were diluted in 1% dimethyl sulfoxide (DMSO) to 1.28 mg/mL (stock solution).

### 2.3. Antifungal Susceptibility

Minimum inhibitory concentration (MIC) assays were performed with the broth microdilution method, as described by the CLSI (Clinical and Laboratory Standards Institute) in documents M27-A3/CLSI [13]. Briefly, 100 *μ*L of each evaluated compound diluted in RPMI 1640 broth was added to 96-well microplates, with the final concentrations of the compounds ranging from 300 to 0.0622 *μ*g/mL (tested substances) and from 32 to 0.065 *μ*g/mL (amphotericin B, antifungal standard). Next, 100 *μ*L of an inoculum containing 2.5 × 10^3^ cells/mL of the reference microorganism was added to 96-well microplates. The microdilution plates were incubated at room temperature (35°C) for 24 to 48 h for *Candida* sp. and *Cryptococcus* spp., respectively. The amount of growth in the tubes containing the tested substance is compared visually with the amount of growth in the growth-control tubes (no antifungal agent) used in each set of tests. In the present work, MIC was defined as the concentration that causes >50% reduction in the growth relative to the drug-free growth control.

### 2.4. Cytotoxicity Assay

The MRC-5 (ATCC-CCL-171-fibroblast/tissue: lung/disease: normal) cell lines were grown in Dulbecco's Modification of Eagle's Medium (DMEM) supplemented with 10% bovine fetal serum, 2 mmol·L^−1^ glutamine, 100 *µ*g/mL streptomycin, and 100 U/mL penicillin and incubated at 37°C with a 5% atmosphere of CO_2_ for 24 h. The substances were individually dissolved in DMSO and added to each well (final concentrations of 0.781–50 *µ*g/mL) and incubated for 72 h. Doxorubicin (5 *µ*g/mL) was used as positive control. Negative controls (blanks) received the same amount of DMSO and had the same final DMSO concentrations as the samples (0.1%). Two hours prior to the end of incubation, 10 *µ*L of AlamarBlue® cell viability reagent was added to each well. The fluorescent signal was monitored with a multiplate reader using a 530–560 nm excitation wavelength range and 590 nm emission wavelength. The fluorescent signal generated from the assay was proportional to the number of living cells in the sample, according to the specifications of the manufacturer [14].

### 2.5. Mechanism of Action Assays

The antifungal mechanism of action of 8-nitroharmane was evaluated using the yeast *C. neoformans* VNI WM148/08 and *C. albicans* strain ATCC 36232 as a model. The influence of 8-nitroharmane on the cell wall (sorbitol protection assay), effect of ergosterol on the cell membrane (ergosterol effect assay), and leakage of substances absorbing at 260 nm were evaluated as described:

#### 2.5.1. Sorbitol Protection Assay

The MIC of 8-nitroharmane was determined against *C. neoformans* VNI and *C. albicans* ATCC 36232 (from 320 to 0.20 *μ*g/mL) in the presence and absence of 0.8 M sorbitol (Sigma-Aldrich) [15, 16]. The MICs were determined after 24 and 72 h of incubation at 35°C.

#### 2.5.2. Ergosterol Effect Assay

The MICs of 8-nitroharmane were determined against *C. neoformans* VNI and *C. albicans* ATCC 36232 (from 320 to 0.20 *μ*g/mL) in the presence (200–800 *μ*g/mL) and absence of exogenous ergosterol (Sigma-Aldrich) [16, 17]. Amphotericin B was used as a control. The MICs were determined after 24 and 72 h of incubation at 35°C. A substance that has affinity for ergosterol rapidly forms complexes with free ergosterol, thus preventing interactions with ergosterol in the fungal membrane; as a result, the MIC of the tested substance increases in the presence of ergosterol [18].

#### 2.5.3. Test for Leakage of Substances Absorbing at 260 nm

*C. neoformans* VNI and *C. albicans* ATCC 36232 were grown in a shaker at 35°C until the early stationary phase (18 h of growth). After incubation, the cells were washed and resuspended in MOPS buffer (0.16 M, pH 7.0). An inoculum of 5 × 10^4^ cells/mL was transferred to microtubes (final volume 500 *μ*L) containing 1 × or 4 MIC of 8-nitroharmane and incubated at 37°C. After 1, 2, 4, or 6 h of incubation, the microtubes were centrifuged (5 min at 3,000 rpm), and the absorbance of the supernatants (100 *μ*L) was measured at 260 nm (Gene Quant DNA/RNA Eppendorf). The absorbance due to leakage from cells treated with HClO_4_ (1.2 M, 100°C, 30 min) was considered 100% [16, 19].

### 2.6. Statistical Analysis

All the values reported in Tables 2–6 are averages of replicate experiments. In general, for a given concentration of a sample or control in the procedures above, replicates had the same experimental value (no variation).

## 3. Results

### 3.1. Screening of Compounds for Anticryptococcal Activity

Of the 11 carbazoles and *β*-carbolines screened, only 8-nitroharmane exhibited significant activity (<50 *µ*g/mL) against *Cryptococcus gattii* VGII and *C. neoformans* VNI (Table 2).

Subsequently, the effects of 8-nitroharmane were investigated on a variety of genotypes of the *Cryptococcus neoformans* and *C. gattii* species complex (Table 3). This compound further exhibited important inhibition (MIC = 40 *μ*g/mL) of genotypes VNIII and VNIV of *C*. *neoformans* and the genotype VGIII of *C*. *gattii*.

### 3.2. Cytotoxicity/Viability Assay

8-nitroharmane did not affect viability of normal human (MRC-5) fibroblasts (IC_50_ > 50 *µ*g/mL) after 24 h of exposure. The cytotoxic standard doxorubicin exhibited IC_50_ = 0.34 (0.27–0.43) *µ*g/mL.

### 3.3. Mechanism of Action Assays

#### 3.3.1. Action on the Cell Wall

A sorbitol protection assay was conducted to determine the influence of 8-nitroharmane on the integrity of the fungal cell wall. In this assay, MIC determinations of 8-nitroharmane against *Cryptococcus neoformans* VNI and *Candida albicans* ATCC 36232 were carried out in parallel in the presence and absence of sorbitol (0.8 M), which is an osmotic protectant used for the stabilization of fungal protoplasts. A substance that interferes negatively with the fungal cell wall will shift the MIC to a higher value in the presence of osmotic support [15]. The MIC of 8-nitroharmane did not change in the presence of sorbitol after 72 h of incubation (Table 4). This is evidence that 8-nitroharmane does not inhibit *Cryptococcus* spp. by interfering with cell walls.

#### 3.3.2. Action on Ergosterol

The ergosterol assay was used to determine whether 8-nitroharmane induces changes in the fungal membrane by interacting with ergosterol. This assay has been shown to identify substances that bind ergosterol in fungal membranes and is based on the addition of exogenous ergosterol. The MIC of 8-nitroharmane did not change in the presence of different concentrations (400 to 1600 *μ*g/mL) of exogenous ergosterol (Table 5). Thus, 8-nitroharmane's inhibition of *Cryptococcus* spp. does not involve interaction with ergosterol. In contrast, a 4-fold increase in the MIC of amphotericin B was observed which is consistent with the strong affinity for ergosterol associated with this substance.

#### 3.3.3. Leakage of Substances Absorbing at 260 nm

The interference of 8-nitroharmane was determined by evaluating the leakage of substances that absorb at 260 nm. Membrane rupture causes the release/leakage of intracellular components from the fungal cell that can then be measured. Nucleotides, which exhibit a strong absorbance at 260 nm, are among the components that can be monitored to detect leakage. 8-nitroharmane (1 × MIC and 4 × MIC) was added to cell suspensions of *C. albicans* and *Cryptococcus neoformans* VNI, and the samples were examined after various time intervals (1, 2, 4, and 6 h). 1 × MIC of 8-nitroharmane caused 6, 9, 17, and 21% increases in cell leakage after 1, 2, 4, and 6 h, respectively, compared to the perchloric acid control (Table 6). Furthermore, the 4 × MIC treatment caused a 2.6 × increase in nucleotide leakage, resulting in 14, 25, 42, and 61% increases in leakage after 1, 2, 4, and 6 h, respectively, compared to perchloric acid (100% cell leakage, control).

## 4. Discussion

Harmine, harmaline, and related *β*Cs are collectively known as harmala alkaloids and were first found in the *Peganum harmala* plant which has many medicinal uses, including the treatment of fungal infections [20]. Harmine and harmaline (isolated from *P*. *harmala*), harmol, harmalol, tetrahydroharmine, and tetrahydroharmol (synthesized from harmine and harmaline) have been screened previously for antifungal activity against 8 dermatophytes, 6 filamentous fungi, and 2 *Candida* spp. [21]. In that work, with the exception of discreet activity against dermatophytes by harmine, the remainder of these compounds were inactive against fungi (MIC > 500 *μ*g/mL). Also according to that work, harmine exhibited MICs of 50 *μ*g/mL against *Trichophyton tonsurans*, 75 *μ*g/mL against *Epidermophyton floccosum*, and 100 *μ*g/mL against *Microsporum canis* and 5 other *Trichophyton* spp. Substances with antifungal properties and with MIC < 100 *µ*g/mL have been suggested as good antifungal agents [22]. Also, as presented in the introduction, harmane [9] and harmine [10] are reported to be potent inhibitors of *Candida albicans*.

To our knowledge, the present study is the first to screen harmala alkaloids, carbazoles, and their derivatives for activity against *Cryptococcus* spp. Herein, no significant inhibitory activity was observed for harmaline, harmane, harmine, harmol, or harmalol against *Cryptococcus* spp. (MIC ≥ 160 *μ*g/mL) in contrast to the activity of these compounds against other fungi according to previous studies presented above. Herein, a new synthetic compound, 8-nitroharmane, that exhibits important inhibition (MIC = 40 *μ*g/mL) of genotypes VNI, VNIII, and VNIV of *C. neoformans* and genotypes VGII and VGIII of *C. gattii* was revealed.

Toxicity is an important factor to be investigated as part of prospecting for new active substances [23]. Concentration-response tests performed with human cells have been increasingly used in the screening of new active substances [24–26]. In experimental conditions herein, 8-nitroharmane did not inhibit normal human cell (MRC-5 fibroblast) cultures even at the highest concentration tested (50 *µ*g/mL) and thus is not cytotoxic [15].

The mode of action of the inhibition of *C. neoformans* and *C. gattii* by 8-nitroharmane was investigated through preliminary testing. This compound was shown neither to act on the cell wall (there was no difference in MIC in the presence or absence of the osmotic protectant sorbitol) nor on ergosterol (there was no difference in MIC in the presence or absence of extracellular ergosterol). However, 8-nitroharmane acted in the extracellular overflow of nucleotides demonstrated by the leakage of nucleic acids from the intracellular medium.

The anticryptococcosis potential of 8-nitroharmane demonstrated in the present work is important. According to the WHO, cryptococcosis should be treated with amphotericin B associated with flucytosine and the fluconazole should be used as a drug of consolidation [3, 27]. However, amphotericin B presents nephrotoxicity in the long-term treatments common to HIV patients. Furthermore, the causal agents of cryptococcosis have demonstrated resistance to azoles such as fluconazole. It is paramount to seek new substances with less adverse effects and tolerable to patients with cryptococcosis [27].

The investigation of new substances with activity against cryptococcosis is important as the current therapeutic arsenal has adverse effects and high toxicity. Other equally toxic medications and the conjugation of this therapy are difficult for patient's tolerance. Herein, the potential of semisynthetic substances derived from *β*-carboline as antifungal agents was demonstrated.

In the present work, 8-nitroharmane was evaluated against *C. neoformans, C. gattii,* and *C. albicans*. In the future work, this compound and more synthetic *β*C derivatives and analogs should be screened against these same as well as other pathogenic *Cryptococcus* spp. Synergistic effects should also be investigated by combining these compounds and in combinations with known fungicides such as amphotericin B or fluconazole against *Cryptococcus* spp. Also, the toxicity of 8-nitroharmane and other *β*Cs to other normal human cell lines should be evaluated as a means to ascertain possible cytotoxic effects not observed in fibroblasts. Further mechanistic studies should be performed to try to ascertain the molecular basis and possible existence of other modes of action. Also, the potential of 8-nitroharmane and other derivatives of harmala alkaloids (*β*Cs) should be evaluated in *in vivo* models of *Cryptococcus* infection as a means to test ethnopharmacological knowledge in systems where metabolism (such as cytochrome P450, known to readily hydroxylate *β*-carbolines) and other interactions with the host may be relevant.

## 5. Conclusion

This work provides a robust account of the screening of eleven substances against two *Cryptococcus* pathogenic species as well as the evaluation of 8-nitroharmane against 8 different genotypes of agents of cryptococcosis. Also, the most active compound 8-nitroharmane exhibited low cellular toxicity to one of the most widely used cell cultures in the literature (MCR-5).

## Figures and Tables

**Table 1:** Names, IUPAC nomenclature, and structures of carbazole and *β*-carboline compounds.

Substance name	Structures	IUPAC name
*N*-acetyl-9H-carbazole	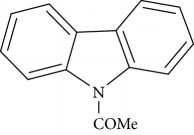	*N*-acetyl-carbazole
9H-carbazole	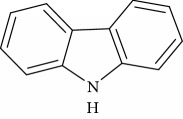	9H-carbazole
Harmaline	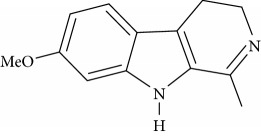	7-Methoxy-1-methyl-4,9-dihydro-3H-pyrido[3,4-b]indole
Harmalol	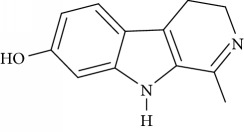	1-Methyl-4,9-dihydro-3H-*β*-carbolin-7-ol
Harmane	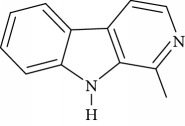	1-Methyl-9H-pyrido[3,4-b]indole
Harmine	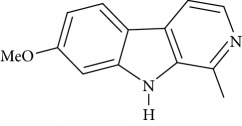	7-Methoxy-1-methyl-9H-pyrido[3,4-b]indole
Harmol	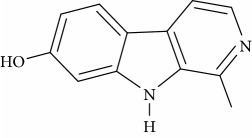	1-Methyl-2,9-dihydropyrido[3,4-b]indol-7-one
6-Nitroharmane	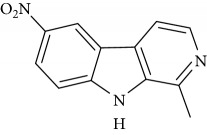	6-Nitro-1-methyl-9H-pyrido[3,4-b]indole
8-Nitroharmane	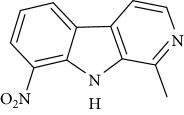	8-Nitro-1-methyl-9H-pyrido[3,4-b]indole
6-Nitroharmine	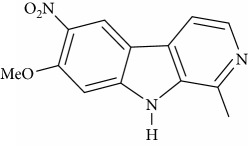	7-Methoxy-6-nitro-1-methyl-9H-pyrido[3,4-b]indole
8-Nitroharmine	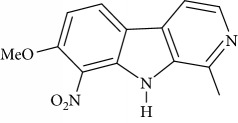	7-Methoxy-8-nitro-1-methyl-9H-pyrido[3,4-b]indole

**Table 2:** MIC of carbazole and *β*-carboline derivatives against *Cryptococcus gattii* and *C. neoformans* strains.

Substances	*C. gattii* VGII WM178/08 MIC (*μ*g/mL)	*C. neoformans* VNI WM148/08 MIC (*μ*g/mL)
8-Nitroharmane	40	40
9H-carbazole	160	160
Harmane	160	160
8-Nitroharmine	160	160
*N*-Acetyl-9H-carbazole	>320	>320
Harmaline	>320	>320
Harmalol	>320	>320
Harmine	>320	>320
Harmol	>320	>320
6-Nitroharmane	>320	>320
6-Nitroharmine	>320	>320
Amphotericin B	0.250	0.065

**Table 3:** MICs of 8-nitroharmane against genotypes of the *Cryptococcus neoformans* and *C. gattii* complex.

Species/molecular types	8-Nitroharmane MIC (*µ*g/mL)	Amphotericin B MIC (*µ*g/mL)
*Candida albicans* ATCC 36232	160	0.5
*C. gattii* VGI WM178/08	80	0.25
*C. gattii* VGIII WM179/08	40	0.25
*C. gattii* VGIV WM779/08	160	0.25
*C. neoformans* VNII WM626/08	80	0.25
*C. neoformans* VNIII WM628/08	40	0.125
*C. neoformans* VNIV WM629/08	40	0.125

**Table 4:** Effect of 0.8 M sorbitol on the MIC of 8-nitroharmane against *Cryptococcus neoformans* and *Candida albicans*.

Substances	*C. neoformans* VNI WM148/08 MIC (*µ*g/mL)		*C. albicans* ATCC 36232 MIC (*µ*g/mL)	
	No sorbitol	0.8 M sorbitol	No sorbitol	0.8 M sorbitol
8-Nitroharmane	40	40	160	160
Amphotericin B	0.065	0.065	0.5	0.5

**Table 5:** Effect of exogenous ergosterol (400–1600 *μ*g/mL) on the MIC of 8-nitroharmane against *Cryptococcus neoformans* and *Candida albicans*.

Substances	*C. neoformans* VNI WM148/08 MIC (*µ*g/mL)				*C. albicans* ATCC 36232 MIC (*µ*g/mL)			
	Control	Ergosterol (*µ*g/mL)			Control	Ergosterol (*µ*g/mL)		
	0	400	800	1600	0	400	800	1600
8-Nitroharmane	40	40	40	40	160	160	160	160
Amphotericin B	0.065	0.065	0.250	0.500	0.5	0.5	1.0	2.0

**Table 6:** Percent leakage of substances absorbing at 260 nm from *Cryptococcus neoformans* and *Candida albicans* induced by 8-nitroharmane.

Percent (%) leakage of substances absorbing at 260 nm									
Substances (concentration)	*C. neoformans* VNI WM148/08				Substances (concentration)	*C. albicans* ATCC 36232			
	1 h	2 h	4 h	6 h		1 h	2 h	4 h	6 h
8-Nitroharmane (40 *µ*g/mL)	0	10	30	60	8-Nitroharmane (160 *µ*g/mL)	0	10	30	60
8-Nitroharmane (320 *µ*g/mL)	10	40	50	60	8-Nitroharmane (640 *µ*g/mL)	10	40	50	60
HClO_4_ (1.2 M)	100	100	100	100	HClO_4_ (1.2 M)	10	100	100	100
Control (blank)	0	0	0	0	Control (blank)	0	0	0	0

## Data Availability

The data used to support the findings of this study are available from the corresponding author upon request.

## Abbreviations

ATCC: American Type Culture Collection
MIC: Minimum inhibitory concentration
SAD: Sabouraud agar dextrose
DMSO: Dimethyl sulfoxide
MOPS: 4-Morpholinepropanesulfonic acid
NCCLS: National Committee for Clinical Laboratory Standards
RPMI: Roswell Park Memorial Institute
